# Rationally Designed Turn Promoting Mutation in the Amyloid-β Peptide Sequence Stabilizes Oligomers in Solution

**DOI:** 10.1371/journal.pone.0021776

**Published:** 2011-07-22

**Authors:** Jayakumar Rajadas, Corey W. Liu, Paul Novick, Nicholas W. Kelley, Mohammed Inayathullah, Melburne C. LeMieux, Vijay S. Pande

**Affiliations:** 1 Biomaterials and Advanced Drug Delivery Laboratory, Stanford University, Stanford, California, United States of America; 2 Department of Chemistry, Stanford University, Stanford, California, United States of America; 3 Stanford Magnetic Resonance Laboratory, Stanford University, Stanford, California, United States of America; 4 Department of Biophysics, Stanford University, Stanford, California, United States of America; 5 Department of Chemical Engineering, Stanford University, Stanford, California, United States of America; 6 Bio-Organic and Neurochemistry Laboratory, Central Leather Research Institute, Chennai, India; Massachusetts Institute of Technology, United States of America

## Abstract

Enhanced production of a 42-residue beta amyloid peptide (Aβ_42_) in affected parts of the brain has been suggested to be the main causative factor for the development of Alzheimer's Disease (AD). The severity of the disease depends not only on the amount of the peptide but also its conformational transition leading to the formation of oligomeric amyloid-derived diffusible ligands (ADDLs) in the brain of AD patients. Despite being significant to the understanding of AD mechanism, no atomic-resolution structures are available for these species due to the evanescent nature of ADDLs that hinders most structural biophysical investigations. Based on our molecular modeling and computational studies, we have designed Met35Nle and G37p mutations in the Aβ_42_ peptide (Aβ_42_Nle35p37) that appear to organize Aβ_42_ into stable oligomers. 2D NMR on the Aβ_42_Nle35p37 peptide revealed the occurrence of two β-turns in the V24-N27 and V36-V39 stretches that could be the possible cause for the oligomer stability. We did not observe corresponding NOEs for the V24-N27 turn in the Aβ_21–43_Nle35p37 fragment suggesting the need for the longer length amyloid peptide to form the stable oligomer promoting conformation. Because of the presence of two turns in the mutant peptide which were absent in solid state NMR structures for the fibrils, we propose, fibril formation might be hindered. The biophysical information obtained in this work could aid in the development of structural models for toxic oligomer formation that could facilitate the development of therapeutic approaches to AD.

## Introduction

The molecular pathology of Alzheimer's disease (AD) is characterized by increased accumulation of 39 to 43 residue long beta-amyloid peptides (Aβ) in plaques in the brains of Alzheimer's disease (AD) patients. Interestingly, individuals of nearly all ages have moderate amounts of matured amyloid peptide fibrils present in their brains, but only a certain percentage of them develop AD. Previous work in this area has indicated that the presence of oligomeric forms (amyloid-derived diffusible ligands, ADDL) of the 42 residue beta-amyloid peptides, rather than fibrils, are responsible for the neuronal damage and synaptic plasticity in the central nervous system in AD [1], [2]. It has also been shown that the severity of neuronal damage is well correlated with the ADDL content of Aβ peptides in the Alzheimer's diseased brain [3].

Monomers of the wild type form of Aβ_42_ (Aβ_42_WT) associate into unstructured assemblies with variable aggregation numbers [4], [5]. Both oligomers and fibrils originate from these unstructured intermediates. Ultimately, ADDLs are unstructured intermediates leaving only matured fibrils as the most stable entities [6], [7]. Much effort has been undertaken to probe the Aβ fibril state utilizing solid-state NMR [8], X-ray [9], Cryo EM [10], electron microscopy [11], neutron scattering [12], atomic force microscopy [13] and other spectroscopic methods [14], [15].

Very little is known about the structure of the ADDL form of the beta-amyloid peptide. The inherent formation of higher order aggregates by Aβ is a great challenge for the experimental characterization of ADDLs. Researchers working in this area are hampered by the difficulties of obtaining sufficient concentrations of ADDL for spectroscopic measurements [16]. Efforts have been made to search for means to stabilize the oligomeric, ADDL, species. For example, Wetzel *et al.* and others have identified small molecules that stabilize the Aβ_42_WT in the proto-fibrillar forms [17], [18]. The work of Selkoe *et al.* [19] comparing neurotoxic properties of Aβ_42_WT and the Arctic mutant E22G Aβs, proposed that the stability of ADDLs is inversely correlated to the nucleation rate of the formation of fibrillar aggregates. Using photo-induced cross-linking and gel electrophoresis, Bitan *et al.* suggested the predominant ADDL species of Aβ_42_WT are monomers, dimers, trimers, pentamers, and hexamers [20]. They have also observed that tetramers are rare and thought to be unstable intermediate species in the oligomerization pathway. A predominantly ADDL forming mutant of Aβ is yet to be known in the literature.

Based on our earlier *in silico* modeling studies, and supported by other work suggesting the formation of β–strand character in the C-terminus of Aβ, we propose an Aβ variant with two point mutations (Met35 to norleucine, and Gly37 to D-proline) to form stable, soluble oligomers [21], [22], [23]. Previous work has demonstrated the ability of single substitutions in the Aβ sequence to alter stability and morphology of Aβ assemblies [24]. First, we substituted the isosteric norleucine in place of Met35 to act as a non-perturbing replacement which would remove issues that could result from the variability of the oxidation state of Met35. Secondly, we substituted a D-proline for G37 in order to stabilize the oligomeric structure seen in simulations, i.e. a C-terminal beta hairpin with a turn at residues 37–38 [23]. In this paper, we probe structural properties of Aβ_42_Nle35p37 and its truncated version Aβ_21–43_Nle35p37 using different biophysical techniques. In particular, we have used Nuclear Magnetic Resonance (NMR) spectroscopy to determine structural properties of ADDL form, and finally use this data in molecular modeling to refine observed structural features.

## Results

### Mutant peptide Aβ_42_Nle35P37 forms stable oligomers in solution

**Figure 1:**
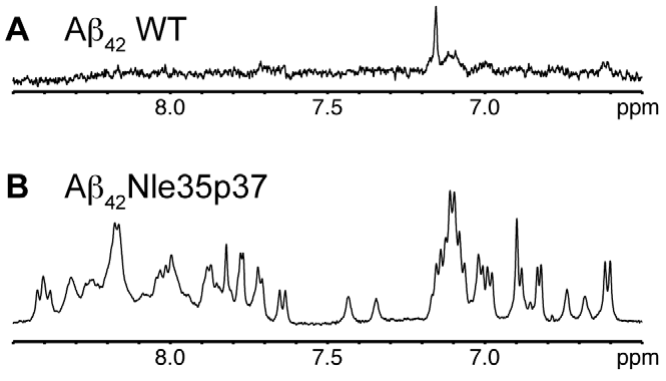
One-dimensional NMR proton spectra of Aβ peptides. Aromatic/amide regions of A) Aβ_42_WT and B) Aβ_42_Nle35p37 in 10% DMSO/PBS, pH 7.2, at 25°C.

In a previous computational study, Kelley *et al* hypothesized that a turn promoting mutation such as G37p would stabilize trimeric Aβ oligomers [23]. In agreement with this prediction, the mutant peptide, Aβ_42_Nle35p37, was found to adopt a significantly higher yield of soluble oligomer than that of Aβ_42_WT. This is seen in the one-dimensional ^1^H NMR spectra of Aβ_42_WT (Figure 1A) and Aβ_42_Nle35p37 (Figure 1B) for samples prepared in the same manner. The Aβ_42_WT spectrum has very little observable signal consistent with the peptide having precipitated from solution (no signal to detect) and/or formed very large molecular weight aggregates (significant line-broadening). Whereas the same spectral region for Aβ_42_Nle35p37 shows amide and aromatic resonances characteristic of soluble, non-aggregating peptides/proteins.

**Figure 2:**
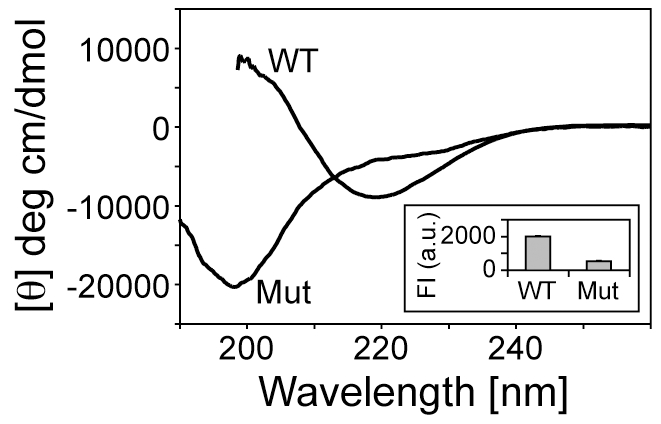
CD spectroscopy of the Aβ_42_Nle35p37 and Aβ_42_WT peptides. Aβ_42_WT takes beta-sheet rich fibrils (curve WT) while Aβ_42_Nle35p37 shows a large negative peak around 197 nm indicating disordered structure (curve Mut). Thioflavin T fluorescence of Aβ_42_WT and Aβ_42_Nle35p37 peptides are shown in the inset. Data was measured at 25°C.

Conformational analyses of the mutant and Aβ_42_WT peptides were also carried out by Circular Dichroism (CD) spectroscopy. A representative CD trace of Aβ_42_WT aggregate is observed to develop into β-sheet rich, mature fibrils over a period of 12 hours with the characteristic strong absorbance at ∼220 nm (Figure 2, Curve WT). However, in the case of the Aβ_42_Nle35p37 mutant, no change was observed in the CD spectrum after one week. Figure 2, Curve *Mut* shows the CD spectrum of Aβ_42_Nle35p37 having a strong absorbance around 197 nm typical of random-coil, disordered states in solution.

Thioflavin T fluorescence was used to assess aggregation and mature fibril formation as β-sheet content is directly correlated with fluorescence intensity of the dye. We observed about four times more Thioflavin T fluorescence in the Aβ_42_WT peptide when compared to Aβ_42_Nle35p37 (Figure 2 inset) confirming the aggregating and non-aggregating nature of the WT and mutant peptides, respectively.

**Figure 3:**
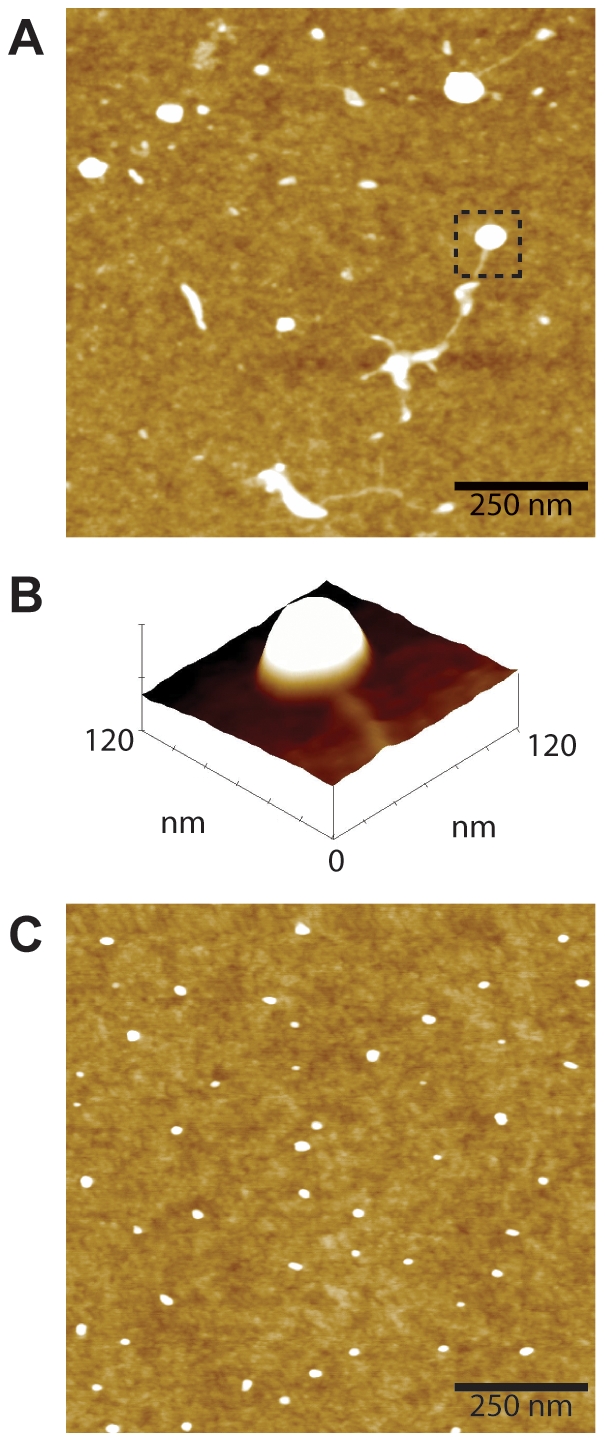
AFM images of Aβ peptide preparations. A) Representative 1.0×1.0-µm x-y, 10-nm total z-range AFM micrograph of Aβ_42_WT preparation. Observed are irregularly shaped and sized aggregate particles, some connected by fibrils. B) A surface plot of the boxed region of (A) clearly showing the aggregate with connected fibril. C) Representative 1.0×1.0-µm x-y, 10-nm total z-range AFM micrograph of Aβ_42_Nle35p37 preparation showing discrete globular aggregates of uniform size and density.

High-resolution analysis of the peptide solution preparations were carried out using atomic force microscopy (AFM). Figure 3 shows AFM images of the soluble form of Aβ_42_WT and Aβ_42_Nle35p37 peptide preparations deposited onto clean silicon wafers. The wafers for Aβ_42_WT wildtype preparations showed particles with a mean globular structure height of 4.32 nm but with a variation of particle sizes ranging from 2.06 to 14.79 nm. Notably, particles appeared to be adhered to each other or connected by thin fibrils suggesting they were in the process of forming larger aggregates. The wafers for the Aβ_42_Nle35p37 mutant preparations showed particles with a similar mean globular structure height of 4.00 nm compared to wildtype, however the distribution of heights was much smaller at 2.29 to 4.84 nm. This, along with the relatively uniform density of globular structures observed, suggests the mutant peptide is stabilized in smaller oligomers.

### Identification of turns and hairpins in Aβ_42_Nle35p37 and Aβ_21–43_Nle35p37 by NMR

**Figure 4:**
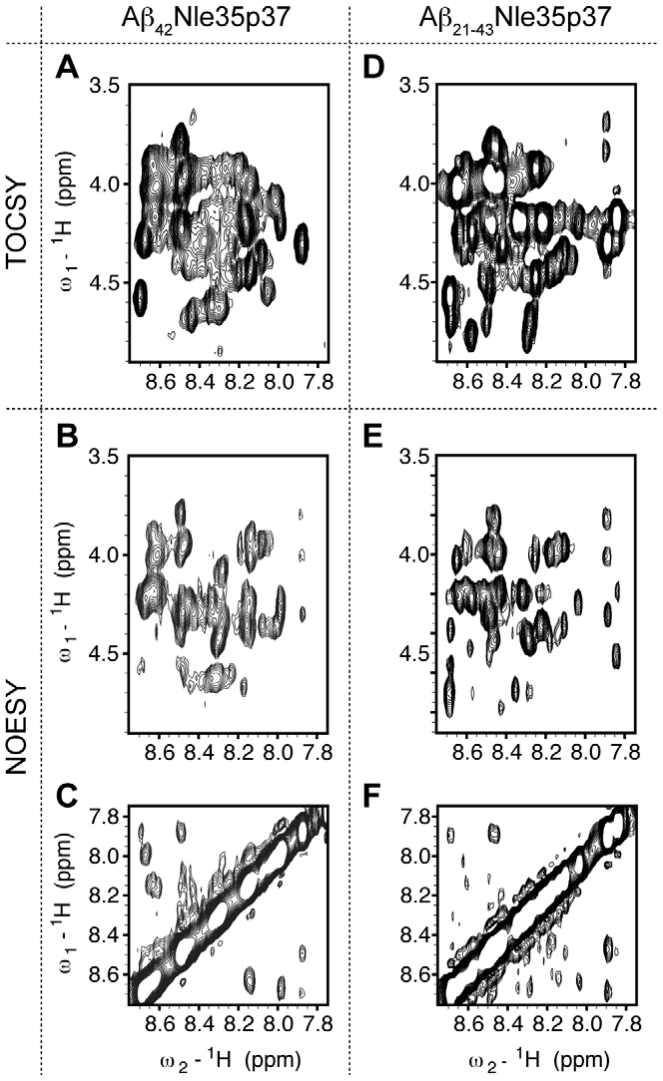
Two-dimensional NMR proton spectra of Aβ peptides. 2D ^1^H-^1^H spectra of Aβ_42_Nle35p37 (A, B, C) and Aβ_21–43_Nle35p37 (D, E, F). TOCSY of (NH-Hα) region of the Aβ_42_Nle35p37 (A) and Aβ_21–43_Nle35p37 (D). NOESY of (NH-Hα) region of Aβ_42_Nle35p37 (B) and Aβ_21–43_Nle35p37 (E). NOESY of (NH-NH) region of Aβ_42_Nle35p37 (C) and Aβ_21–43_Nle35p37 (F). Data was measured at 15°C in 10% DMSO-d_6_, PBS, pH 7.2.

Having demonstrated that the full-length mutant Aβ_42_Nle35p37 peptide forms assumes a stable, low-molecular weight form in solution we proceeded with multi-dimensional NMR experiments on the mutant peptide. The quality of two-dimensional ^1^H-^1^H TOCSY and NOESY spectra were marginal due to signal overlap and line-broadening (Figure 4ABC) likely caused by a combination of low-level aggregation, conformational heterogeneity, and conformational averaging between transiently structured conformations.

In order to aid the assignment problem, we built on previous work suggestive that the N-terminal residues of the Aβ sequence are flexible and unstructured by working with a truncated form of the mutant peptide, Aβ_21–43_Nle35p37 [23], [25]. When HFIP film of this truncated mutant peptide was dissolved, we observed higher solubility (>4 mg/ml) compared to the full-length mutant peptide (∼1 mg/ml). This truncated peptide remained in solution without any fibril formation, similar to the full-length mutant, for a period of more than 6 months (data not shown).

The TOCSY and NOESY spectra of the truncated peptide gave slightly better quality data (Figure 4DEF) when compared to the full-length mutant peptide. Assignments, though not trivial, were more tractable on this construct. Working with the information gleaned from the truncated form allowed a degree of validation for the full-length mutant assignments (the spectral profiles showed similarities though not one-for-one overlays). Interestingly, save for Y10 and K16, the N-terminal residues in the full-length mutant peptide, Aβ_42_Nle35p37, were not readily assigned, consistent with the N-terminus being flexible and unstructured. Assignments are given in Tables 1 and 2 for Aβ_42_Nle35p37 and Aβ_21–43_Nle35p37, respectively.

**Table 1:** Chemical shift assignments of Aβ_42_Nle35p37.

Amino acid	NH	αH	βH	Others
Y10	8.02	4.554	2.95, 3.08	
K16	8.242	4.317	1.554, 1.662	δH 1,405
E22	8.18	4.355	2.218	
D23	8.349	4.672	2.685	
V24	8.379	4.292	1.806	γH 0.9618
G25	8.143	4.01		
S26	8.203	3.927	4.484	
N27	8.339	4.616	3.063	
K28	8.475	3.97	-	εH 2.293
G29	8.64	3.898		
A30	8.082	4.366	1.406	
I31	8.165	4.196	1.909	εH 0.9618
I32	8.627	4.17	1.9	εH 0.9591
G33	8.602	4.026		
L34	8.143	4.445	1.627	-
NL35	8.301	4.63	1.616	γH1.227, εH1.105
V36	8.7	4.583	2.021	γH 0.9852
dP37		4.22	2.39	γH 2.081, 2.143
				δH 3.885
G38	8.494	3.791		
V39	7.88	4.3	2.12	γH 0.94
V40	8.488	4.235	2.248	γH 0.9513
I41	8.678	4.291	2.016	εH 0.981
A42	7.989	4.36	1.401	

**Table 2:** Chemical shift assignments of Aß_21–43_Nle35p37.

Amino acid	NH	αH	βH	others
E22	8.543	4.743	1.92	γH 1.268
D23	8.47	4.455	2.795	
V24	8.275	4.17	1.873	γH 0.926
G25	8.418	3.788		
S26	8.214	3.9	4.451	
N27	8.644	4.661	2.682	
K28	8.464	4.665	1.978,1.885	γH 1.423 εH 2.8
G29	8.616	3.993		
A30	8.068	4.333	1.374	
I31	8.179	4.172	1.874	γH 1.202 εH 0.889
I32	8.54	4.218	1.91	εH 0.93
G33	8.464	3.949		
L34	8.141	4.428	1.642	δH 0.9494
NL35	8.242	4.675	1.606	
V36	8.648	4.537	2.023	γH 0.957
dP37		4.423	2.373	γH 2.006 , 2.1
				δH 3.892
G38	8.401	3.97		
V39	7.853	4.274	2.88	γH 0.92
V40	8.446	4.487	1.98	γH 0.9118
I41	8.314	4.168	2.191	γH 0.9655
A42	8.442	4.178	1.418	

A wealth of significant NOE crosspeaks was not expected from these peptides but the few that we observed were rather interesting. Comparative intensities of NOEs for Hα-NH (i, i+1) and Hα-NH (i, i) are indicative of turn conformation [26], [27], [28]. Specifically, residues that have extended conformation show higher Hα-NH (i, i+1) NOE intensity compared to that of Hα-NH (i, i), while residues involved in turn conformation show higher Hα-NH (i, i) intensity than the corresponding Hα-NH (i, i+1) intensity. For the full length mutant Aβ_42_Nle35p37 peptide, Hα-NH (i,i) NOE cross peaks are observed for G25-S26 and G38-V39. Also observed are NH-NH (V24-G25) and NH-NH (G25-S26) NOEs suggesting a type I turn in the region of V24-N27. Hα-NH (p37-G38) and NH-NH (G38-V39) NOEs suggest that the p37-G38 segment forms a type II′ β-turn. The Hα-Hδ (V36-p37) cross peak observed between V36 and p37 indicates that p37 is in the *trans* conformation. Earlier work on Aβ_42_WT monomers suggests turns around D7-Y10 and V24-N27 [29], [30]. Our data supports the existence of this second turn. Though we do not observe the first turn, we cannot discount the possibility of its presence being very transient in nature given the difference in spectral properties observed between the full-length and truncated mutant peptides.

For the truncated mutant Aβ_21–43_Nle35p37 peptide, as in the full-length mutant, we observed Hα-NH (p37-G38) and NH-NH (G38-V39) NOE cross peaks suggesting a type II′ β-turn involving residues V36-V39. Similarly, an Hα-Hδ (V36, p37) cross peak suggests the presence of trans conformation for p37. We do not observe NH-NH NOEs for the turn around V24-N27, but this may be the result of N-terminal flexibility in this truncated construct.

### Results from computational structure refinement

**Figure 5:**
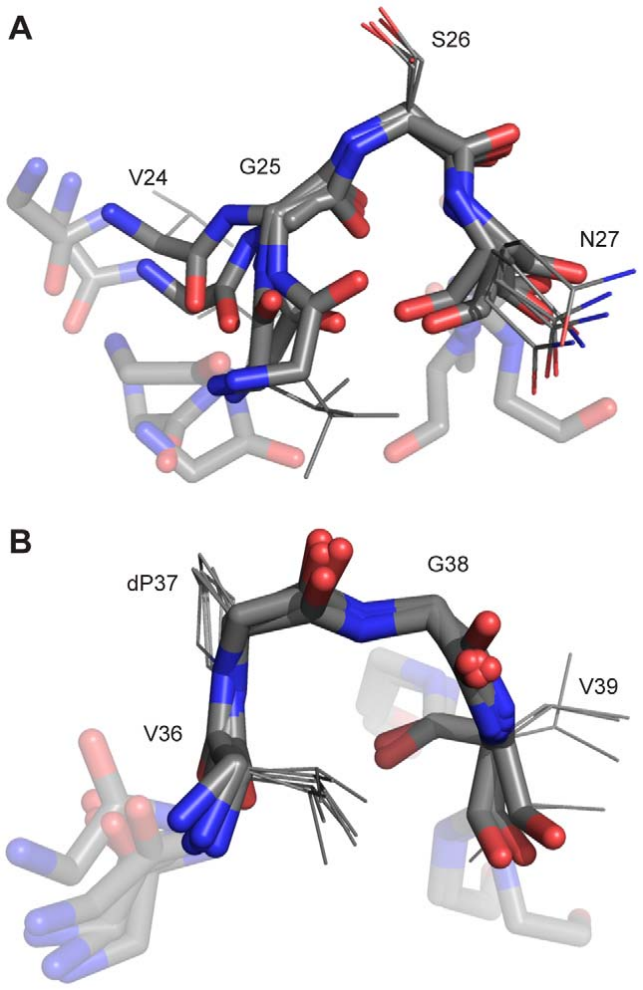
NOE refinement ensembles. 5 structures were taken at 1 ns intervals from the computational structure refinements. Shown are the six residues around the turns (solid color rendering for the four residues around the turns, semi-transparent for the leading and trailing residues), backbone heavy-atoms shown for all residues, and side-chain heavy-atoms included for the four residues around the turns. (A) The V24-N27 turn is observed in most SS-NMR studies, and as show here has a conformation similar to previous unconstrained MD simulations. (B) The induced beta-turn from the d-Pro mutation, V36-V39, is clearly defined.

Refinement via molecular dynamics using the NMR constraints was used to produce an ensemble of structures for the NOE containing regions of the full length mutant Aβ_42_Nle35p37 peptide. (Figure 5, PDB S1 and PDB S2) We see the existence of two definite turns – the D-proline induced beta-hairpin (residues V36-V39) and a more N-terminal turn consisting of residues V24-N27, which is in agreement with the available SS-NMR fibril studies and complimentary unconstrained MD simulations [6], [31], [32], [33].

### Mutant peptide decreases fibril content of WT aggregate mixtures

**Figure 6:**
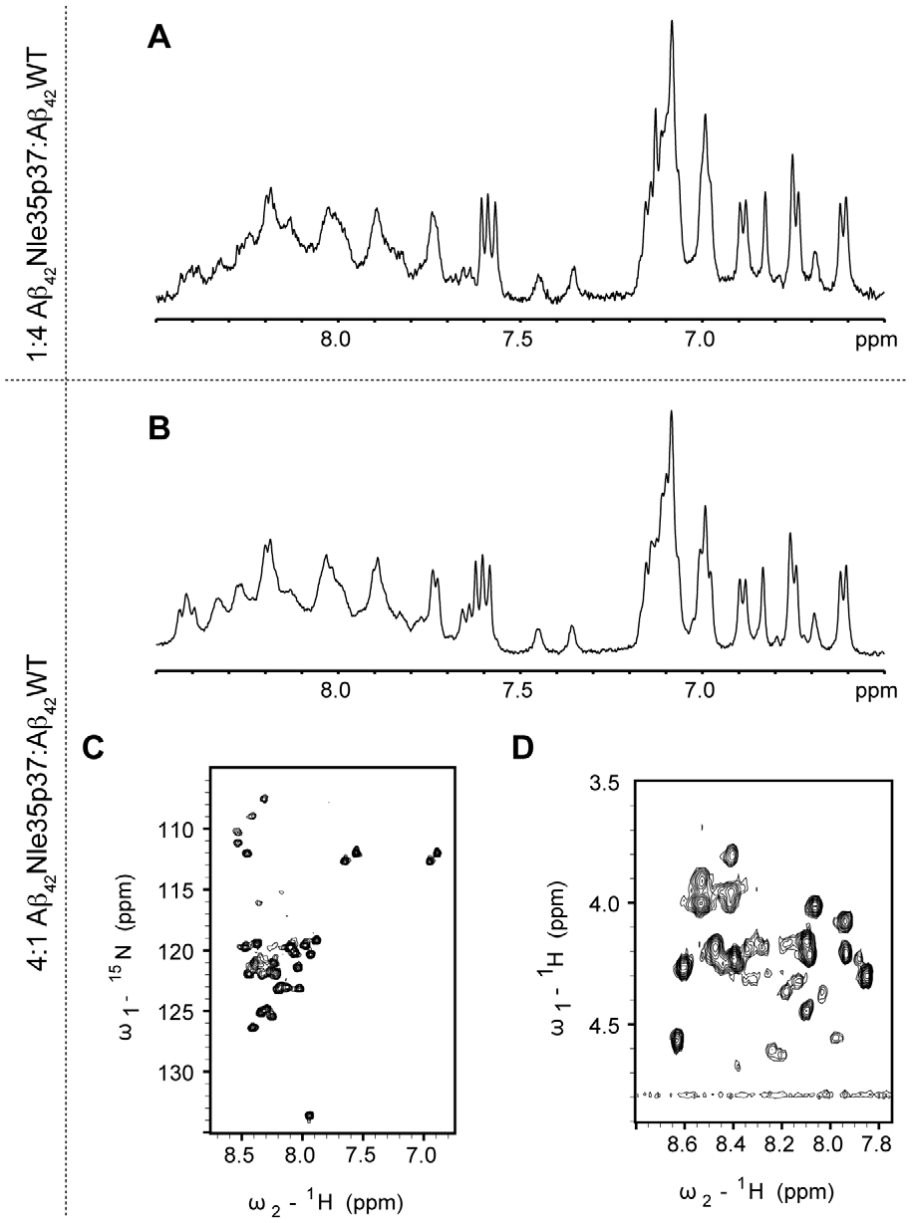
One- and two-dimensional NMR spectra of Aβ peptide mixtures. 1D proton spectra of the aromatic/amide regions of 1∶4 (A) and 4∶1 (B) mixtures of Aβ_42_Nle35p37∶Aβ_42_WT. (C) 2D ^1^H-^15^N HSQC (Heteronuclear Single Quantum Coherence) experiment of the 4∶1 Aβ_42_Nle35p37∶Aβ_42_WT mixture (Aβ_42_WT uniformly ^15^N-labeled), in 10% DMSO/PBS, pH 7.2, at 25°C. (D) 2D ^1^H-^1^H TOCSY (NH-Hα) region of the 4∶1 Aβ_42_Nle35p37∶Aβ_42_WT mixture, in 10% DMSO/PBS, pH 7.2.

Aβ_42_WT was found to adopt a soluble oligomeric form to a much greater extent when mixed with the mutant. By co-solubilizing Aβ_42_WT with Aβ_42_Nle35p37 in 1∶4 and 4∶1 ratios, the mixtures resulted in one-dimensional ^1^H NMR spectra very similar to that acquired on Aβ_42_Nle35p37 alone (Figure 6AB). To ensure the spectra observed were not simply the result of all Aβ_42_WT precipitating, oligomerizing, or aggregating to itself leaving only mutant Aβ_42_Nle35p37 peptide observable by NMR, we also produced the same WT: mutant peptide mixtures using uniformly ^15^N-labeled Aβ_42_WT. We were able to observe resolved ^1^H{^15^N} HSQC spectra (Figure 6C) verifying the ability of the mutant peptide stabilizing the WT in solution, and suggesting that the mutant peptide could act as an inhibitor to fibril formation of Aβ_42_WT. Acquiring two-dimensional TOCSY experiments on the mixture samples found similarities in CαH chemical shifts to the mutant peptide alone (Figure 6D) suggestive that the Aβ_42_WT adopts a similar conformation to the Aβ_42_Nle35p37 mutant when the two are mixed.

## Discussion

In our effort to design a soluble oligomer-forming mutant, we have previously predicted that the mutation of G37p will result in enhanced β hairpin formation and increased oligomer stability. Two-turn structures were obtained using NOE refinement. The occurrence of the first turn at V24-N27 is in agreement with the available SS-NMR fibril studies. The second turn consisting of residues V36-V39, absent in previous studies, is introduced here by the mutation. In the nucleated polymerization model fibrils are likely to grow by monomer addition [34]. The two-turn structure of the mutant possibly depletes monomer content by diverting peptide into a stable oligomer formation.

Earlier NMR studies showed that Aβ monomers adopts a collapsed coil (mostly random) with a well-defined central hydrophobic cluster (L17-A21) and turn- or bend-like structures (D7-E11 and A21-S26) [35]. The data presented here are consistent with these previous observations. Additionally, we have shown a β-turn in the C-terminal region of the peptide. Wuthrich *et al.* have also studied the structure of the oxidized form of Met35^ox^ Aβ_40_ and Aβ_42_ peptides in aqueous Tris-HCl buffered solutions at pH 6.4–8.2 [36]. They showed unstructured peptide strands punctuated by turns around S8-V12 and F20-V24 regions. Their ^15^N{^1^H} NOE data showed that the Aβ_42_Met35^ox^ has reduced flexibility at the C-terminus relative to the Aβ_40_ Met35^ox^ suggesting insipient structure around this region, consistent with our hypothesis of a beta hairpin in the same region.

How does this structural data compare to previous SS-NMR data studies on fibril structure? Almost all fibrillar studies show a hairpin turn forming somewhere between residues 24 and 30. Examining three of the most recent and notable studies, we find three different sets of amino acids forming the turn location, although it is arguable that this may be attributed to the differences in information content for the various methods and their corresponding sensitivity to structural disorder [6]. Ohman's 2006 study of residues 1–42 predict a turn consisting of residues 25–28 or GSNK in the sequence [31]. Riek's predicted turn of the same chain is shifted two amino acids towards the N-terminus at residues 27–30 and sequence NKGA [32]. Tycko's recent work concerning Aβ_40_WT has found a turn at residues V24-N27 with sequence VGSN [6]. This turn location is in agreement with our oligomer data. Moreover, our ensemble highly resembles an unconstrained MD study of the same chain, in which the ensemble was clustered and the most populated node was presented [33].

Our refined ensemble shows a less static ensemble than the SS-NMR fibril studies. The d-Pro induced turn at residues V36-V39 disrupts the inter-chain contacts present in the fibril models by changing the monomer topology. This leaves the VGS turn sequence to stabilize itself exclusively via intra-chain contacts, and we suggest this to be the reason we see greater flexibility in this region.

The AFM data for the mutant Aβ_42_Nle35p37 peptide suggest that it forms predominantly low molecular weight species in solution. The 4 nm mean AFM particle height would be in the range of five- to eight-mer complexes by a statistical analysis performed by Lobanov *et al.* on the radius of gyration of >3500 protein domains in the SCOP database [37]. But notably, the domains in the Lobanov study were compact, folded, α and/or β containing proteins. The likelihood of the Aβ mutant peptide being in a loose, predominantly undefined structure, would possibly reduce the number of monomer units present per complex.

### Conclusions

The data presented in this paper indicate the structurally disordered oligomeric assemblages of Aβ_42_WT and mutant differ in their propensity to form oligomers and fibrils. Aβ_42_WT peptide formed fibrils at the concentration of 0.4 mM at 10% DMSO/ PBS. The mutant preparation resulted entirely in low molecular weight entities. NMR studies on Aβ_42_Nle35p37 showed occurrence of two β-turns in the stretches V24-N27 and V36-V39.

Upon mixing Aβ_42_Nle35p37 mutant with Aβ_42_WT, Aβ_42_WT peptide is stabilized in solution suggesting a significant reduction in fibril formation. Presumably such reduced fibril formation is due to the engineered β-turn of the mutant (V36-V39) hindering the formation of the C-terminal β-turn (V24-A30) found in the fibril SS-NMR structure. Although our finding implies the existence of a stabilizing structure for the ADDLs in the mutant peptide, we were not able to detect any known secondary structure stretches, other than the two β-turns, by ^1^H-NMR and CD spectroscopy. This suggests that β-sheet or α-helix formation is not required for the ADDL stability. Finally, the ability of this mutant to inhibit the aggregation of WT Aβ peptide opens a door to another use for this mutant peptide, since a variant of this peptide or a small molecule peptide mimic could potentially serve as a means to inhibit Aβ aggregation.

How are these results useful in gaining insight into the nature of WT Aβ? While our NMR structural data of the mutant does not directly give structural data regarding the WT, the fact that the mutant mixed with the WT has slowed aggregation suggests that the C terminal beta hairpin presumably stabilized by the mutant does have structural relevance for understanding the nature of the aggregation of WT Aβ. Future work could either use the C terminal beta hairpin motif for small molecule drug discovery in order to find novel small molecule inhibitors of Aβ aggregation.

## Materials and Methods

### Sample preparation

Synthetic peptide Aβ_42_Nle35p37 of the sequence DAEFRHDSGY^10^EVHHQKLVFF^20^AEDVGSNKGA^30^IIGL^N^LV^D^pGVV^40^IA was synthesized and purified by Anaspec (San Jose, CA). AEDVGSNKGA^30^IIGL^N^LV^D^pGVV^40^IAT (Aβ_21–43_Nle35p37) was synthesized and purified by the Stanford Protein and Nucleic Acid Facility. Recombinant purified Aβ_42_WT, both unlabeled and ^15^N-labeled, peptides (>95%) were obtained from rPeptide (Bogart, GA). All peptides were used as supplied. Solution samples of the peptides were prepared as follows. Peptides were dissolved in 1,1,1,3,3,3-hexafluoro-2-propanol (HFIP) to concentrations of 1 mM HFIP, evaporated over nitrogen, and then dried in a Savant Speed Vac for 1 hr. The resultant peptide films were further kept under vacuum for a few hours to remove solvent traces. The films were stored at −80°C until use. Peptide films were dissolved to ∼0.5 mM concentrations in 10% d_6_-DMSO/10 mM PBS buffer to a final pH of 7.2. The solutions were incubated at 4°C for 12 hours, and then transferred to 37°C for 4 hours. The resulting solutions were centrifuged at 13000 rpm in a desktop centrifuge (Eppendorf) at 4°C for 30 min to remove any precipitated gel-form of the peptides. After centrifugation, the peptide concentrations were adjusted to 0.4 mM (concentrations were checked by UV visible spectroscopy), 0.05% sodium azide added as a bacteriostat and transferred to 5 mm NMR tubes. Mixtures (4∶1 and 1∶4) of Aβ_42_Nle35p37∶Aβ_42_WT (total peptide concentration of 1 mM) were prepared in HFIP. The solvent was evaporated using speed-vac and resultant peptide films were dissolved in 10% d_6_-DMSO/10 mM PBS buffer and processed as above. d_6_-DMSO was purchased from Cambridge Isotope Laboratories (Cambridge, MA). Tris(2,2′-bipyridyl)dichloro ruthenium(II) (Ru(Bpy)) and ammonium persulfate (APS) were purchased from Sigma.

### Circular Dichroism Spectroscopy (CD) and Thioflavin T binding studies

Aβ_42_Nle35p37, having been dissolved and processed above (10% DMSO/PBS buffer), was passed through a 10/30 Superdex 75 HR column to remove DMSO and immediately analyzed by CD. An Aβ_42_WT sample was prepared into PBS buffer in order to demonstrate a representative beta-sheet rich CD trace. The fibrils from the Aβ_42_WT preparation were centrifuged at 12000 rpm for 15 min to separate from any remaining monomer and the pellet was re-suspended in 10 mM phosphate pH 7.2 for CD measurement. CD measurements were performed using a 0.1 mm path length quartz cell in an Aviv 62A DS Circular Dichroism spectrometer (Aviv Associates, Lakewood, NJ) at 22°C.

Mean residue ellipticity (θ) was determined according to the equation θ = θ_Obs _• MRW/(10 • *l* • *c*); where θ_Obs_ is observed ellipticity, MRW is mean residue weight of the peptide, *c* is peptide concentration (g/L), and *l* is optical path length (cm). The thioflavin T (ThT) binding studies of Aβ_42_WT and mutant peptides were carried out by incubating precipitated fibrils of the Aβ_42_WT or eluted oligomer preparation from the SEC column. The 25 µM amyloid fibrils (Aβ_42_WT) or oligomers of mutant were mixed with 5 µM of ThT dye concentration. After 2 h incubation, the fluorescence of the sample solutions were recorded by exciting at 440 nm and the intensity at 480 nm is monitored using Perkin-Elmer LC 55B spectrofluorimeter. The bandwidths of excitation/emission wavelengths were 5 nm/each.

### Atomic Force Microscopy

AFM topography images were acquired in the light tapping mode regime using a Multimode AFM (Veeco, USA). Resonant frequencies of the uncoated silicon tips (MikroMasch, USA) were roughly 150 kHz, with scan rates around 5–8 µm/s. Peptide samples were prepared using the protocol described above and diluted to 25 µM before application. Peptide solutions were adsorbed by incubating onto Pirahna cleaned silicon wafers with molecular smoothness (∼1 Å RMS roughness) as determined by AFM. After incubating for 10 min, the wafers are gently washed repeatededly with MilliQ water.

### NMR Spectroscopy

NMR spectra were acquired at the Stanford Magnetic Resonance Laboratory on a Bruker Avance 500 MHz spectrometer running TopSpin v1.3 and equipped with a 5 mm H{CN} Z-axis gradient CryoProbe. Peptide samples were prepared as described above in 10% d_6_-DMSO/10 mM PBS buffer of pH 5.5 or 7.2. One-dimensional ^1^H experiments were acquired with 16384 total data points, 12 ppm spectral window, and number of scans ranging from 64 to 256. Two-dimensional ^1^H-^1^H TOCSY (TOtal Correlation SpectroscopY) experiments were acquired with 2048 total data points, 11 ppm spectral windows, 60 or 80 ms dipsi2 mixing times, for 256 to 512 increments of 32 to 128 scans per increment. Two-dimensional ^1^H-^1^H NOESY (Nuclear Overhauser Effect SpectroscopY) experiments were acquired with 2048 total data points, 11 ppm spectral windows, 100, 150, 200 or 400 ms mixing times, for 128 increments of 128 scans per increment. The two-dimensional ^1^H-^15^N HSQC (Heteronuclear Single Quantum Coherence) experiment was acquired with 1024 total data points, 12 ppm (^1^H) and 40 ppm (^15^N) spectral windows, for 128 increments of 256 scans per increment. Sample temperatures were regulated from 15 to 30°C. Water suppression was accomplished with WATERGATE in each experiment. The data was processed in TopSpin and analyzed with SPARKY [38].

### Computational Structure Refinement

Atomic level structures for the 2 predicted turns were obtained using molecular dynamics. Chains of eight amino acids long at the site of each turn were created in an extended coil conformation. Each chain was capped with an acetyl group on the N-terminus and a N-methyl on the C-terminus to avoid any termini association due to charge effects. Chains were then solvated with tip3p water in a dodecahedral box, with dimensions allowing a 1.5 nm separation between peptide atoms and the edge of the box. After an energy minimization, a harmonic potential was added based on the NOE data. The spring constant for this potential was set to 1250 kJ/mol, and applied outside of the accepted range of 0.5 nm, with a truncation creating a constant maximum force beyond 0.6 nm. NOE distances were allowed to fluctuate and updated every 10 ps, allowing the capability for multiple constraints on each atom. Forces contributing to the hydrogen-bond distances were applied at every step for a more rigid network. The peptides were simulated using Gromacs and the Amber 2003 force field [39], [40], [41], [42]. After 3 ns equilibration time, each system was run for 5 ns with structures taken every 1 ns, producing an ensemble.

## Supporting Information

PDB S1 PDB file of five snapshots of the V24-N27 turn from NMR refinement, as displayed in Figure 5a.
(PDB)

PDB S2 PDB file of five snapshots of the V36-V38 turn from NMR refinement, as displayed in Figure 5b.
(PDB)
